# Bis(3-amino­propan-1-aminium) naphthalene-1,5-disulfonate dihydrate

**DOI:** 10.1107/S1600536812018296

**Published:** 2012-04-28

**Authors:** Shan Gao, Seik Weng Ng

**Affiliations:** a Key Laboratory of Functional Inorganic Material Chemistry, Ministry of Education, Heilongjiang University, Harbin 150080, People’s Republic of China; b Department of Chemistry, University of Malaya, 50603 Kuala Lumpur, Malaysia; c Chemistry Department, Faculty of Science, King Abdulaziz University, PO Box 80203 Jeddah, Saudi Arabia

## Abstract

In the title hydrated salt, 2C_3_H_11_N_2_^+^·C_10_H_6_O_6_S_2_^2−^·2H_2_O, the anion lies on a center of inversion; its sulfonate –SO_3_ group features one S—O bond that is longer than the other two. The O atom of this longer bond is the hydrogen-bond acceptor to the amino H atom of one cation and the ammonium H atom of another cation. In the crystal, N—H⋯O and O—H⋯O hydrogen bonds link the cations, anions and water mol­ecules into a three-dimensional network.

## Related literature

For a related structure, see: Gao & Ng (2012 ▶). 
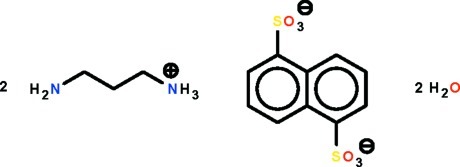


## Experimental

### 

#### Crystal data

2C_3_H_11_N_2_^+^·C_10_H_6_O_6_S_2_^2−^·2H_2_O
*M* *_r_* = 472.58
Triclinic, 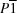

*a* = 8.0913 (16) Å
*b* = 8.1917 (16) Å
*c* = 9.8062 (18) Å
α = 71.182 (5)°
β = 65.950 (5)°
γ = 79.945 (5)°
*V* = 561.12 (19) Å^3^
*Z* = 1
Mo *K*α radiation
μ = 0.29 mm^−1^
*T* = 293 K
0.21 × 0.17 × 0.17 mm

#### Data collection

Rigaku R-AXIS RAPID IP diffractometer
Absorption correction: multi-scan (*ABSCOR*; Higashi, 1995 ▶) *T*_min_ = 0.942, *T*_max_ = 0.953
5584 measured reflections
2552 independent reflections
2268 reflections with *I* > 2σ(*I*)
*R*_int_ = 0.039

#### Refinement

*R*[*F* ^2^ > 2σ(*F* ^2^)] = 0.044
*wR*(*F* ^2^) = 0.127
*S* = 1.05
2552 reflections
165 parameters
7 restraints
H atoms treated by a mixture of independent and constrained refinement
Δρ_max_ = 0.44 e Å^−3^
Δρ_min_ = −0.39 e Å^−3^

### 

Data collection: *RAPID-AUTO* (Rigaku, 1998 ▶); cell refinement: *RAPID-AUTO*; data reduction: *CrystalClear* (Rigaku/MSC, 2002 ▶); program(s) used to solve structure: *SHELXS97* (Sheldrick, 2008 ▶); program(s) used to refine structure: *SHELXL97* (Sheldrick, 2008 ▶); molecular graphics: *X-SEED* (Barbour, 2001 ▶); software used to prepare material for publication: *publCIF* (Westrip, 2010 ▶).

## Supplementary Material

Crystal structure: contains datablock(s) global, I. DOI: 10.1107/S1600536812018296/xu5521sup1.cif

Structure factors: contains datablock(s) I. DOI: 10.1107/S1600536812018296/xu5521Isup2.hkl

Supplementary material file. DOI: 10.1107/S1600536812018296/xu5521Isup3.cml

Additional supplementary materials:  crystallographic information; 3D view; checkCIF report

## Figures and Tables

**Table 1:** Hydrogen-bond geometry (Å, °)

*D*—H⋯*A*	*D*—H	H⋯*A*	*D*⋯*A*	*D*—H⋯*A*
O1w—H1w1⋯O1	0.84 (1)	2.00 (1)	2.826 (2)	166 (3)
O1w—H1w2⋯N1	0.84 (1)	1.91 (1)	2.736 (2)	172 (3)
N1—H12⋯O3^i^	0.88 (1)	2.55 (2)	3.317 (2)	147 (2)
N2—H21⋯O1w^ii^	0.89 (1)	1.84 (1)	2.715 (2)	172 (2)
N2—H22⋯O2^iii^	0.89 (1)	1.98 (1)	2.845 (2)	165 (3)
N2—H23⋯O3^iv^	0.88 (1)	1.97 (1)	2.825 (2)	164 (2)

Symmetry codes: (i) 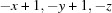; (ii) 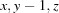; (iii) 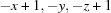; (iv) 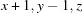.

**Fig. 1.**
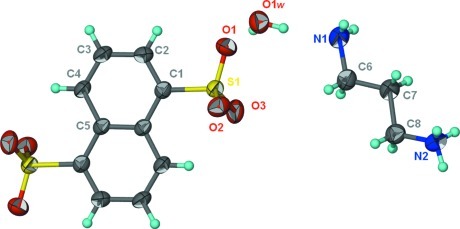
Thermal ellipsoid plot (Barbour, 2001) of 2(C3H11N2)2+.(C10H6O6S2)2-.2H2O at the 70% probability level; hydrogen atoms are drawn as spheres of arbitrary radius. Symmetry-related atoms are not labeled.

## supplementary crystallographic information

### Comment

The study continues with the preceding study on ammonium naphthalene-1,5-disulfonates (Gao & Ng, 2012). The synthesis of the propane-1,3-diammonium derivative yielded instead the bis(3-aminopropylammonium) salt, which exists as a dihydrate (Scheme I), *i.e.*, only one of the two amino groups is protonated so that the cation has a monopositive charge only. The anion of 2(C_3_H_11_N_2_)^2+.^(C_10_H_6_O_6_S_2_)^2-.^2H_2_O lies on a center-of-inversion (Fig. 1). The sulfonate –SO_3_ group features one S–O bond that is longer than the other two; the O atom of this longer bond is hydrogen-bond acceptor two the amino H atom of one cation and the ammonium H atom of another cation. Hydrogen bonds link the cation, anion and water molecule into a three-dimensional network.

### Experimental

A methanol solution (5 ml) of 1,3-propanediamine (98% pure, 1 mmol, 0.08 ml) was added to an aqueous solution (5 ml) of 1,5-naphthalenedisulfonic acid tetrahydrate (0.5 mmol, 180 mg). The mixture heated at 343 K until the reacctants dissolved completely; colorless crystals were isolated from the filtrate after several days. three days.

### Refinement

Carbon-bound H-atoms were placed in calculated positions (C–H 0.93 Å) and were included in the refinement in the riding model approximation, with *U*(H) set to 1.2*U*(C). The amino and water H-atoms were located in a difference Fourier map, and were refined with distance restraints N–H 0.88±0.01 Å, O–H 0.84±0.01 Å and H···H 1.37±0.01 Å; their temperature factors were refined.

### Crystal data

**Table:**

2C_3_H_11_N_2_^+^·C_10_H_6_O_6_S_2_^2^^−^·2H_2_O	*Z* = 1
*M**_r_* = 472.58	*F*(000) = 252
Triclinic, *P*1	*D*_x_ = 1.399 Mg m^−^^3^
Hall symbol: -P 1	Mo *K*α radiation, λ = 0.71073 Å
*a* = 8.0913 (16) Å	Cell parameters from 3803 reflections
*b* = 8.1917 (16) Å	θ = 3.0–27.5°
*c* = 9.8062 (18) Å	µ = 0.29 mm^−^^1^
α = 71.182 (5)°	*T* = 293 K
β = 65.950 (5)°	Prism, colorless
γ = 79.945 (5)°	0.21 × 0.17 × 0.17 mm
*V* = 561.12 (19) Å^3^	

### Data collection

**Table:**

Rigaku R-AXIS RAPID IP diffractometer	2552 independent reflections
Radiation source: fine-focus sealed tube	2268 reflections with *I* > 2σ(*I*)
Graphite monochromator	*R*_int_ = 0.039
ω scan	θ_max_ = 27.5°, θ_min_ = 3.0°
Absorption correction: multi-scan (*ABSCOR*; Higashi, 1995)	*h* = −10→9
*T*_min_ = 0.942, *T*_max_ = 0.953	*k* = −10→10
5584 measured reflections	*l* = −12→12

### Refinement

**Table:**

Refinement on *F*^2^	Secondary atom site location: difference Fourier map
Least-squares matrix: full	Hydrogen site location: inferred from neighbouring sites
*R*[*F*^2^ > 2σ(*F*^2^)] = 0.044	H atoms treated by a mixture of independent and constrained refinement
*wR*(*F*^2^) = 0.127	*w* = 1/[σ^2^(*F*_o_^2^) + (0.0736*P*)^2^ + 0.1592*P*] where *P* = (*F*_o_^2^ + 2*F*_c_^2^)/3
*S* = 1.05	(Δ/σ)_max_ < 0.001
2552 reflections	Δρ_max_ = 0.44 e Å^−^^3^
165 parameters	Δρ_min_ = −0.39 e Å^−^^3^
7 restraints	Extinction correction: *SHELXL97* (Sheldrick, 2008), Fc^*^=kFc[1+0.001xFc^2^λ^3^/sin(2θ)]^-1/4^
Primary atom site location: structure-invariant direct methods	Extinction coefficient: 0.081 (12)

### Fractional atomic coordinates and isotropic or equivalent isotropic displacement parameters (Å^2^)

**Table:**

	*x*	*y*	*z*	*U*_iso_*/*U*_eq_	
S1	0.27701 (5)	0.72860 (5)	0.28439 (4)	0.02880 (18)	
O1	0.40860 (17)	0.78029 (17)	0.12668 (14)	0.0406 (3)	
O2	0.35741 (17)	0.62846 (16)	0.39549 (15)	0.0402 (3)	
O3	0.12666 (17)	0.64501 (17)	0.29413 (17)	0.0425 (3)	
O1W	0.75757 (19)	0.63049 (19)	0.10446 (16)	0.0421 (3)	
H1W1	0.6524 (18)	0.661 (3)	0.106 (3)	0.057 (7)*	
H1W2	0.766 (4)	0.5307 (17)	0.096 (3)	0.063 (8)*	
N1	0.8221 (3)	0.3040 (2)	0.0702 (2)	0.0437 (4)	
H11	0.9387 (16)	0.288 (3)	0.053 (3)	0.063 (8)*	
H12	0.792 (4)	0.294 (4)	−0.003 (2)	0.067 (8)*	
N2	0.7492 (2)	−0.3142 (2)	0.36576 (18)	0.0367 (4)	
H21	0.744 (3)	−0.340 (3)	0.287 (2)	0.059 (7)*	
H22	0.701 (3)	−0.400 (3)	0.450 (2)	0.065 (7)*	
H23	0.8614 (16)	−0.323 (3)	0.361 (3)	0.046 (6)*	
C1	0.1830 (2)	0.9236 (2)	0.33721 (18)	0.0269 (3)	
C2	0.2538 (2)	1.0749 (2)	0.23475 (18)	0.0320 (4)	
H2	0.3496	1.0745	0.1411	0.038*	
C3	0.1822 (2)	1.2322 (2)	0.27039 (19)	0.0341 (4)	
H3	0.2318	1.3352	0.2003	0.041*	
C4	0.0411 (2)	1.2350 (2)	0.40653 (18)	0.0297 (4)	
H4A	−0.0057	1.3403	0.4274	0.036*	
C5	−0.03570 (19)	1.08034 (19)	0.51689 (17)	0.0251 (3)	
C6	0.7250 (3)	0.1771 (2)	0.2140 (2)	0.0387 (4)	
H6A	0.5962	0.1970	0.2356	0.046*	
H6B	0.7462	0.1951	0.2985	0.046*	
C7	0.7781 (3)	−0.0097 (2)	0.2119 (2)	0.0385 (4)	
H7A	0.7530	−0.0300	0.1302	0.046*	
H7B	0.9073	−0.0301	0.1882	0.046*	
C8	0.6774 (2)	−0.1352 (2)	0.3650 (2)	0.0360 (4)	
H8A	0.6896	−0.1063	0.4488	0.043*	
H8B	0.5496	−0.1262	0.3825	0.043*	

### Atomic displacement parameters (Å^2^)

**Table:**

	*U*^11^	*U*^22^	*U*^33^	*U*^12^	*U*^13^	*U*^23^
S1	0.0281 (3)	0.0244 (2)	0.0307 (3)	−0.00188 (15)	−0.00813 (18)	−0.00714 (17)
O1	0.0406 (7)	0.0360 (7)	0.0315 (6)	0.0011 (5)	−0.0028 (5)	−0.0076 (5)
O2	0.0405 (7)	0.0329 (7)	0.0413 (7)	0.0000 (5)	−0.0168 (6)	−0.0019 (5)
O3	0.0366 (7)	0.0390 (7)	0.0580 (8)	−0.0049 (5)	−0.0135 (6)	−0.0256 (6)
O1W	0.0412 (7)	0.0395 (8)	0.0456 (8)	0.0018 (6)	−0.0141 (6)	−0.0169 (6)
N1	0.0528 (10)	0.0322 (8)	0.0420 (9)	−0.0036 (7)	−0.0147 (8)	−0.0085 (7)
N2	0.0376 (8)	0.0327 (8)	0.0368 (8)	−0.0065 (6)	−0.0146 (7)	−0.0028 (6)
C1	0.0298 (7)	0.0245 (8)	0.0278 (7)	−0.0035 (6)	−0.0122 (6)	−0.0063 (6)
C2	0.0339 (8)	0.0307 (9)	0.0262 (7)	−0.0073 (6)	−0.0066 (6)	−0.0047 (6)
C3	0.0418 (9)	0.0238 (8)	0.0311 (8)	−0.0107 (7)	−0.0097 (7)	−0.0011 (6)
C4	0.0368 (8)	0.0210 (8)	0.0297 (8)	−0.0063 (6)	−0.0110 (7)	−0.0043 (6)
C5	0.0273 (7)	0.0233 (8)	0.0265 (7)	−0.0050 (6)	−0.0123 (6)	−0.0046 (6)
C6	0.0396 (9)	0.0340 (9)	0.0424 (9)	−0.0042 (7)	−0.0133 (8)	−0.0123 (8)
C7	0.0425 (9)	0.0321 (9)	0.0390 (9)	−0.0020 (7)	−0.0143 (8)	−0.0087 (7)
C8	0.0337 (8)	0.0347 (9)	0.0395 (9)	−0.0041 (7)	−0.0125 (7)	−0.0106 (7)

### Geometric parameters (Å, º)

**Table:**

S1—O1	1.4484 (13)	C2—H2	0.9300
S1—O2	1.4484 (13)	C3—C4	1.362 (2)
S1—O3	1.4532 (13)	C3—H3	0.9300
S1—C1	1.7848 (16)	C4—C5	1.416 (2)
O1W—H1W1	0.840 (10)	C4—H4A	0.9300
O1W—H1W2	0.837 (10)	C5—C5^i^	1.427 (3)
N1—C6	1.457 (2)	C5—C1^i^	1.436 (2)
N1—H11	0.883 (10)	C6—C7	1.520 (2)
N1—H12	0.879 (10)	C6—H6A	0.9700
N2—C8	1.479 (2)	C6—H6B	0.9700
N2—H21	0.886 (10)	C7—C8	1.508 (2)
N2—H22	0.885 (10)	C7—H7A	0.9700
N2—H23	0.881 (9)	C7—H7B	0.9700
C1—C2	1.360 (2)	C8—H8A	0.9700
C1—C5^i^	1.436 (2)	C8—H8B	0.9700
C2—C3	1.408 (2)		
			
O1—S1—O2	113.11 (8)	C3—C4—C5	121.11 (15)
O1—S1—O3	111.96 (8)	C3—C4—H4A	119.4
O2—S1—O3	112.63 (8)	C5—C4—H4A	119.4
O1—S1—C1	106.07 (7)	C4—C5—C5^i^	119.01 (17)
O2—S1—C1	106.32 (8)	C4—C5—C1^i^	123.18 (14)
O3—S1—C1	106.10 (7)	C5^i^—C5—C1^i^	117.81 (16)
H1W1—O1W—H1W2	100 (2)	N1—C6—C7	114.59 (16)
C6—N1—H11	108.1 (17)	N1—C6—H6A	108.6
C6—N1—H12	107.7 (18)	C7—C6—H6A	108.6
H11—N1—H12	115 (3)	N1—C6—H6B	108.6
C8—N2—H21	110.4 (16)	C7—C6—H6B	108.6
C8—N2—H22	119.3 (18)	H6A—C6—H6B	107.6
H21—N2—H22	107 (2)	C8—C7—C6	112.28 (15)
C8—N2—H23	108.1 (15)	C8—C7—H7A	109.1
H21—N2—H23	111 (2)	C6—C7—H7A	109.1
H22—N2—H23	101 (2)	C8—C7—H7B	109.1
C2—C1—C5^i^	121.35 (15)	C6—C7—H7B	109.1
C2—C1—S1	118.04 (13)	H7A—C7—H7B	107.9
C5^i^—C1—S1	120.60 (12)	N2—C8—C7	110.76 (14)
C1—C2—C3	120.06 (15)	N2—C8—H8A	109.5
C1—C2—H2	120.0	C7—C8—H8A	109.5
C3—C2—H2	120.0	N2—C8—H8B	109.5
C4—C3—C2	120.65 (15)	C7—C8—H8B	109.5
C4—C3—H3	119.7	H8A—C8—H8B	108.1
C2—C3—H3	119.7		
			
O1—S1—C1—C2	−4.68 (15)	S1—C1—C2—C3	179.05 (12)
O2—S1—C1—C2	115.97 (14)	C1—C2—C3—C4	−0.5 (3)
O3—S1—C1—C2	−123.91 (14)	C2—C3—C4—C5	1.0 (3)
O1—S1—C1—C5^i^	174.63 (12)	C3—C4—C5—C5^i^	−0.7 (3)
O2—S1—C1—C5^i^	−64.71 (14)	C3—C4—C5—C1^i^	179.21 (15)
O3—S1—C1—C5^i^	55.41 (14)	N1—C6—C7—C8	178.34 (16)
C5^i^—C1—C2—C3	−0.3 (2)	C6—C7—C8—N2	−172.59 (15)

Symmetry code: (i) −*x*, −*y*+2, −*z*+1.

### Hydrogen-bond geometry (Å, º)

**Table:**

*D*—H···*A*	*D*—H	H···*A*	*D*···*A*	*D*—H···*A*
O1w—H1w1···O1	0.84 (1)	2.00 (1)	2.826 (2)	166 (3)
O1w—H1w2···N1	0.84 (1)	1.91 (1)	2.736 (2)	172 (3)
N1—H12···O3^ii^	0.88 (1)	2.55 (2)	3.317 (2)	147 (2)
N2—H21···O1w^iii^	0.89 (1)	1.84 (1)	2.715 (2)	172 (2)
N2—H22···O2^iv^	0.89 (1)	1.98 (1)	2.845 (2)	165 (3)
N2—H23···O3^v^	0.88 (1)	1.97 (1)	2.825 (2)	164 (2)

Symmetry codes: (ii) −*x*+1, −*y*+1, −*z*; (iii) *x*, *y*−1, *z*; (iv) −*x*+1, −*y*, −*z*+1; (v) *x*+1, *y*−1, *z*.
